# Impact of bileaflet mitral valve prolapse on quantification of mitral regurgitation with cardiac magnetic resonance: a single-center study

**DOI:** 10.1186/s12968-017-0362-6

**Published:** 2017-07-27

**Authors:** Gabriella Vincenti, Pier Giorgio Masci, Tobias Rutz, Jonathan De Blois, Milan Prša, Xavier Jeanrenaud, Juerg Schwitter, Pierre Monney

**Affiliations:** 1 Center for Cardiac Magnetic Resonance (CRMC), University Hospital of Lausanne (CHUV), Lausanne, Switzerland; 2 Pediatric Cardiology unit, University Hospital of Lausanne (CHUV), Lausanne, Switzerland; 3 Service de Cardiologie, Département Cœur - Vaisseaux, University Hospital of Lausanne (CHUV), Lausanne, Switzerland

**Keywords:** Mitral regurgitation, Mitral valve, Prolapse, Barlow, Cardiac magnetic resonance

## Abstract

**Background:**

To quantify mitral regurgitation (MR) with CMR, the regurgitant volume can be calculated as the difference between the left ventricular (LV) stroke volume (SV) measured with the Simpson’s method and the reference SV, i.e. the right ventricular SV (RVSV) in patients without tricuspid regurgitation. However, for patients with prominent mitral valve prolapse (MVP), the Simpson’s method may underestimate the LV end-systolic volume (LVESV) as it only considers the volume located between the apex and the mitral annulus, and neglects the ventricular volume that is displaced into the left atrium but contained within the prolapsed mitral leaflets at end systole. This may lead to an underestimation of LVESV, and resulting an over-estimation of LVSV, and an over-estimation of mitral regurgitation. The aim of the present study was to assess the impact of prominent MVP on MR quantification by CMR.

**Methods:**

In patients with MVP (and no more than trace tricuspid regurgitation) MR was quantified by calculating the regurgitant volume as the difference between LVSV and RVSV. LVSV_uncorr_ was calculated conventionally as LV end-diastolic (LVEDV) minus LVESV. A corrected LVESV_corr_ was calculated as the LVESV plus the prolapsed volume, i.e. the volume between the mitral annulus and the prolapsing mitral leaflets. The 2 methods were compared with respect to the MR grading. MR grades were defined as absent or trace, mild (5–29% regurgitant fraction (RF)), moderate (30–49% RF), or severe (≥50% RF).

**Results:**

In 35 patients (44.0 ± 23.0y, 14 males, 20 patients with MR) the prolapsed volume was 16.5 ± 8.7 ml. The 2 methods were concordant in only 12 (34%) patients, as the uncorrected method indicated a 1-grade higher MR severity in 23 (66%) patients. For the uncorrected/corrected method, the distribution of the MR grades as absent-trace (0 vs 11, respectively), mild (20 vs 18, respectively), moderate (11 vs 5, respectively), and severe (4 vs 1, respectively) was significantly different (*p* < 0.001). In the subgroup without MR, LVSV_corr_ was not significantly different from RVSV (difference: 2.5 ± 4.7 ml, *p* = 0.11 vs 0) while a systematic overestimation was observed with LVSV_uncorr_ (difference: 16.9 ± 9.1 ml, *p* = 0.0007 vs 0). Also, RVSV was highly correlated with aortic forward flow (*n* = 24, *R* ^2^ = 0.97, *p* < 0.001).

**Conclusion:**

For patients with severe bileaflet prolapse, the correction of the LVSV for the prolapse volume is suggested as it modified the assessment of MR severity by one grade in a large portion of patients.

## Background

Mitral regurgitation (MR) is one of the most common valve diseases with an estimated prevalence of 1.7% in the general population, increasing to 9.3% after age 75. Moderate to severe MR represents an important public health issue as it is associated with poor clinical outcome [1]. Degenerative mitral valve prolapse (MVP) is considered as one of the most common causes of MR in the general population [2] and it accounts for 60 to 70% of cases in surgical series [3]. MVP is defined as a systolic excursion of the mitral leaflets >2 mm behind the mitral annular plane in long axis view, i.e. a displacement of >2 mm into the left atrium (LA) [4].

In general, for the quantification of MR severity echocardiography is the method of choice, although some limitations exist. For example, the most frequently used technique, the proximal isovelocity surface area (PISA) method [5], may be technically challenging in mitral valve prolapse patients as the effective regurgitant orifice may vary during systole [6, 7], potentially leading to inaccurate grading of severity. A recent study suggested that cardiovascular magnetic resonance (CMR) might be superior to echocardiography in identifying MR patients, who will benefit most from mitral surgery in terms of post-operative left ventricular (LV) remodelling [8]. By accurately measuring volumes and flows [9], CMR is an attractive method to quantify mitral regurgitant volume (Reg_Vol_) and regurgitant fraction (RF), two parameters that may be able to identify patients suitable for early surgery [10]. Several techniques have been validated for Reg_Vol_ calculation, which are based on the difference between the LV stroke volume (SV) measured with the Simpson’s method and a reference SV either measured as the aortic forward flow (Ao_forward_) by phase-contrast cine CMR at the level of the ascending aorta or as the right ventricular (RV) SV by the Simpson’s method (assuming the absence of significant associated aortic, pulmonary or tricuspid regurgitation) [11–18].

With the method of disks, the end-diastolic and end-systolic volumes are measured by tracing the endocardial border of consecutive short-axis cine slices covering the LV from the apex to the mitral annulus. For patients with prominent MVP, this strategy may be inaccurate in systole as the part of the LV volume contained within the prolapsing mitral leaflets (ie behind the mitral annulus) at end-systole is simply neglected and excluded from the total LV end-systolic volume (and erroneously considered as being part of the left atrial volume). Thus, the currently accepted approaches [11–18] do not take into account the anatomical features of MVP. We hypothesized that this LVESV underestimation leads to an overestimation of the LVSV and hence, to an overestimation of MR severity in MVP patients. Similarly, in patients with MVP but without MR, the corrected LVSV should be in agreement with the reference stroke volume of the RV (i.e. it should avoid a LVSV vs RVSV difference) or aortic forward flow volume. The amount of error of standard, uncorrected LVESV measurements is expected to be proportional to the height of the valve prolapse, i.e. to the “prolapsed volume”.

Accordingly, in this study, we proposed a simple strategy to correct the LVESV for the severity of the prolapse, i.e. for the “prolapsed volume”, and we assessed its impact on the quantification of MR severity in patients with significant MVP.

## Methods

### Patient population

A retrospective search was conducted in our CMR centre between January 2011 and January 2017 to identify the patients, for which the presence of bileaflet MVP could be suspected. The keywords “prolapse”, “billowing”, “Barlow”, and “Marfan” were used to screen the CMR reports and 105 patients were identified. The images were individually reviewed to only include patients presenting with bileaflet MVP, defined as the billowing of both, the anterior and posterior mitral leaflets, with displacement of the leaflets >2 mm into the LA on a 3 chamber long-axis view in systole [4]. The clinical records were analyzed for the presence of aortic, mitral, tricuspid or pulmonary regurgitation detected by any other means (usually echocardiography). Patients were excluded if they had more than mild regurgitation of the aortic or pulmonary valve, more than trace regurgitation of the tricuspid valve, or any intracardiac shunt.

### CMR acquisition

Image acquisition was performed on a 1.5 T (Magnetom AERA or Magnetom Symphony) or a 3.0 T (Magnetom Verio or Magnetom Skyra) scanner (Siemens Healthcare, Erlangen Germany) using a 32-channels phased-array coil. A balanced steady-state free precession cine sequence was used for ventricular volume assessment (typical parameters TR 3.06 ms, TE 1.28 ms, flip angle 60°, FOV 300 × 240 mm, voxel size 1.2 × 1.2 × 8.0 mm, 16 lines/segment, 25 phases/cardiac cycle). A stack of short axis slices (slice thickness 8 mm and 2 mm gap) from the base to the apex, as well as 3 long-axis slices of the LV, were acquired in all patients. When clinically indicated, a phase-contrast acquisition of the ascending aorta was additionally performed during breath-hold (typical parameters TR 10.5 ms, TE 3.04 ms, flip angle 20°, FOV 340 × 238 mm, voxel size 1.8 × 1.8 × 6.0 mm, 4 lines/segment, 20 phases/cardiac cycle, VENC 150 cm/s); the imaging plane for aortic forward flow measurement was defined from two orthogonal long axis cine acquisitions of the ascending aorta and was placed at the level of the right pulmonary artery.

### Image analysis

**Fig. 1:**
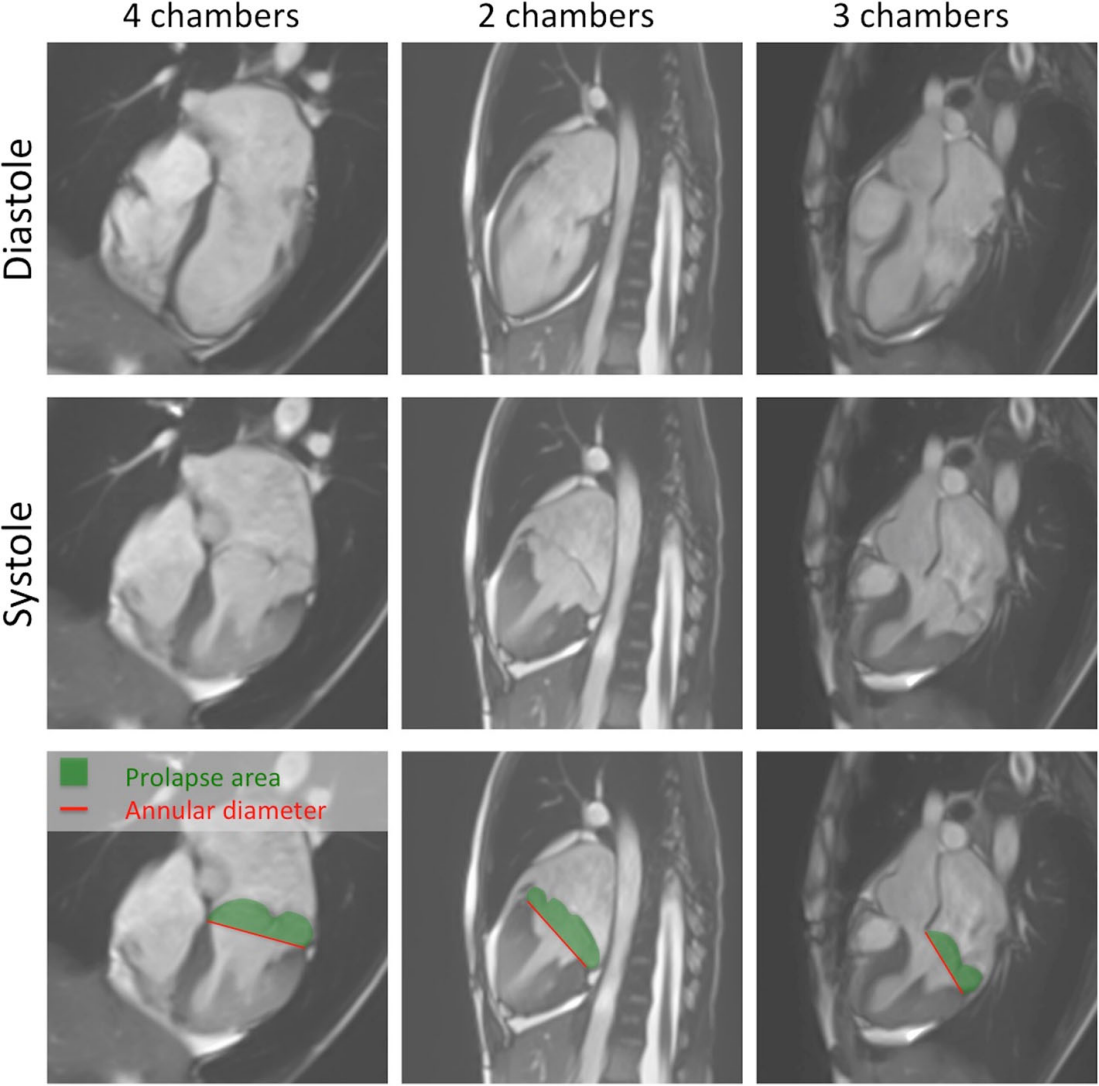
Severe bileaflet mitral valve prolapse in a patient with Marfan syndrome. *Top row*: diastolic frames in 4-, 2- and 3-chamber orientations. *Mid row*: corresponding systolic frames. *Bottom row*: on each systolic view, the prolapsed area is measured by planimetry (*green area*). This area is divided by the mitral annular diameter (*red line*) to calculate the prolapsed height. The average of the three calculated prolapsed heights gives the mean prolapsed height of the mitral valve

**Fig. 2:**
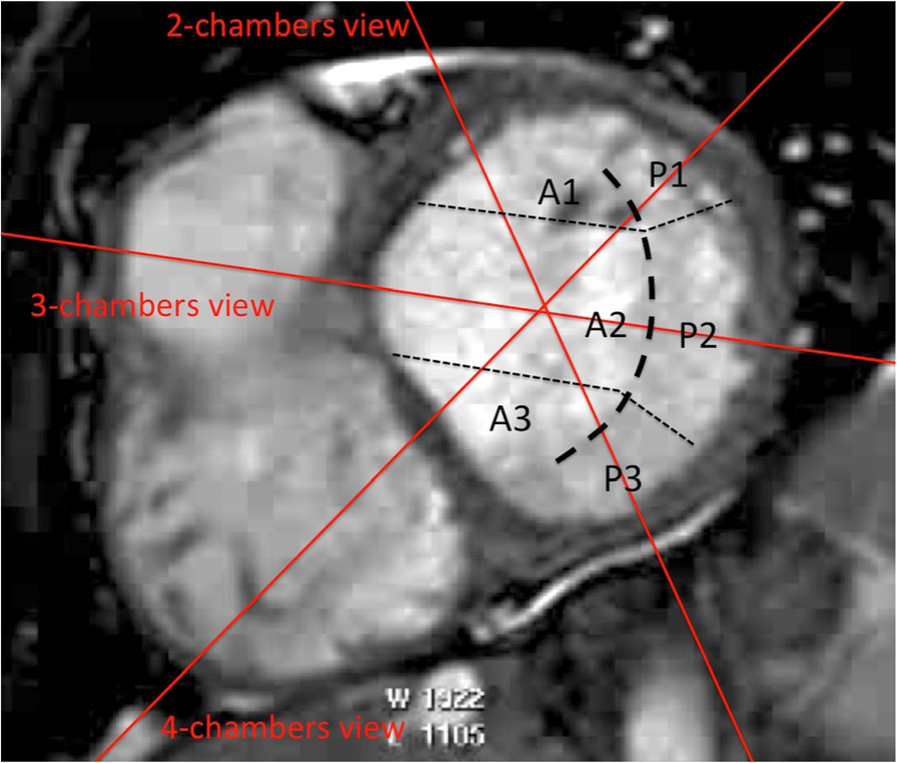
Morphology of the mitral valve according to the Carpentier’s classification. The posterior mitral leaflet is naturally separated by cleft like indentations into 3 scallops (P1, P2 and P3). The corresponding segments of the anterior leaflet are called A1, A2 and A3. The long axis 3-chamber view will cut the A2 and P2 scallops of the mitral valve, the 2-chamber view the A1, A2 and P3 scallops, and the 4-chamber view the P1, A2 and A3 scallops

The ventricular volumes were measured according to the Simpson’s method of disks by tracing the endocardial LV border from the basal to the apical slice using a semi-automated border detection software (GTvolume®, Gyrotools, Zürich, Switzerland). Muscular trabeculae were included in the blood pool. For end-diastolic (LVEDV) and uncorrected end-systolic (LVESV_uncorr_) LV volumes, the basal slice was defined as the slice with LV myocardium detected at least 50% of its circumference [19, 20]. In case of uncertainty, cross-referencing of basal short axis and long axis views was used. A prolapse-corrected LV end-systolic volume (LVESV_corr_) was additionally measured as the standard LVESV (=LVESV_uncorr_) plus the prolapsed volume. The prolapsed volume was calculated as the area of the basal end-systolic slice multiplied by the mean prolapsed height. The mean prolapsed height was the average of the three individual prolapsed heights of the three standard LV long-axis views of the LV (ie 2-, 3-, and 4-chamber views), calculated as the prolapsed area divided by the annulus diameter (Fig. 1). This strategy aimed to obtain a better coverage of the mitral valve and its 6 different scallops (Fig. 2) to more accurately estimate the prolapsed volume as patients not infrequently present with asymmetrical prolapses or prolapses predominantly involving the posterior mitral leaflet. The uncorrected LV stroke volume (LVSV_uncorr_) was calculated as the LVEDV minus the LVESV_uncorr_. The prolapse-corrected stroke volume (LVSV_corr_) was calculated as the LVEDV minus LVESV_corr_.

The RV volumes were measured from the same set of short-axis cine images in all patients according to the Simpson’s method of disks by tracing the endocardial RV border from the basal to the apical slice using the same semi-automated border detection software. Cross-referencing with a long axis four-chamber cine acquisition was performed in all patients to accurately identify the basal RV slice. The RVSV was calculated as the difference between the end-diastolic and end-systolic RV volume and was considered the reference stroke volume in view of the fact that tricuspid insufficiency was a study exclusion criteria. When available, the aortic forward flow (Ao_forward_) corrected for background phase was measured on phase-contrast cine images of the ascending aorta by manually tracing the aortic surface frame-by-frame using the Argus workstation (Siemens Healthcare, Erlangen, Germany).

For the assessment of MR severity, the regurgitant volume was calculated as the difference between the LVSV_uncorr_ and the RVSV (= standard or uncorrected method) and between the LVSV_corr_ and the RVSV (= corrected method). For both methods, the RF was calculated as the ratio between the respective regurgitant volume and the respective LVSV. A RF <5% defined absent or trace MR, 5–29% mild MR, 30–49% moderate MR, and ≥50% severe MR [21].

### Statistical analysis

Continuous variables were expressed as mean ± standard deviation and categorical variables as percentages. The correlations and agreements between uncorrected and corrected LVSV were analyzed by linear regression and Bland-Altman statistics [22]. Direct comparisons of baseline characteristics were performed with paired *t*-tests. Direct comparison between LVSV and the reference stroke volume were performed with Wilcoxon matched-pairs signed ranks test. The comparison of the MR grade distribution was performed with Chi-square test. All calculations were performed with Stata 14 software (StataCorp LP, Texas, USA). A *p*-value <0.05 was considered as significant.

## Results

### Patient population

Forty-two patients were identified with bi﻿﻿lea﻿flet ﻿MVP. One patient was excluded due to the presence of an atrial septal defect with significant left-to-right shunt and six patient had mild or moderate tricuspid regurgitation. Thirty-five patients were therefore included in the analysis. Mean age was 44.0 ± 23.0 years and 14 (40%) were male. Fifteen patients (42.9%) with MVP had no or only trace MR detected on echocardiography, or no MR jet detected on the 3 long axis or basal short-axis cine CMR acquisitions. Additional baseline characteristics are summarized in Table 1.

**Table 1:** Patients characteristics

	All patients (*n* = 35)	No MR (*n* = 15)	Significant MR (*n* = 20)	*p*
Age (years)	44.0 ± 23.0	38.1 ± 19.1	48.5 ± 25.1	0.19
Height (cm)	174.5 ± 12.4	178.1 ± 13.2	171.9 ± 11.4	0.14
Weight (kg)	62.4 ± 15.9	64.5 ± 18.8	60.8 ± 13.7	0.5
BMI (kg/m^2^)	20.3 ± 3.8	20.1 ± 4.3	20.4 ± 3.4	0.82
LVEDV (ml)	155.4 ± 51.1	151.5 ± 58.3	158.4 ± 46.3	0.7
LVEDV index (ml/m^2^)	88.5 ± 22.8	83.4 ± 22.6	92.2 ± 22.8	0.27
LVESV uncorrected (ml)	53.3 ± 23.3	50.9 ± 25.6	55.0 ± 22.0	0.62
LVESV corrected (ml)	69.8 ± 28.4	65.3 ± 29.7	73.1 ± 27.8	0.43
LVEF standard (%)	66.4 ± 7.3	67.2 ± 7.1	65.7 ± 7.6	0.56
LVEF corrected (%)	55.6 ± 8.3	57.6 ± 7.2	54.1 ± 8.9	0.22
Prolapsed height (mm)	8.1 ± 2.8	7.6 ± 2.1	8.5 ± 3.2	0.32
Prolapsed volume (ml)	16.5 ± 8.7	14.4 ± 7.0	18.1 ± 9.6	0.22
RVEDV (ml)	140.1 ± 46.3	157.4 ± 55.6	127.2 ± 33.8	0.05
RVESV (ml)	68.6 ± 24.5	73.8 ± 28.7	64.7 ± 20.9	0.29
RVEF (%)	51.0 ± 6.6	53.5 ± 5.3	49.1 ± 7.0	0.05
Indication for CMR (n; %)				
• Marfan syndrome	11 (31)	6 (40)	5 (25)	0.02
• MR quantification	9 (26)	0 (0)	9 (45)	
• Ventricular arrhythmia	7 (20)	6 (40)	1 (5)	
• Ischemia detection	5 (14)	2 (13)	3 (15)	
• Suspicion of myocarditis	1 (3)	0 (0)	1 (5)	
• Malformation syndromes	2 (6)	1 (7)	1 (5)	

### Reference stroke volume

RVSV was measured in all patients with a mean value of 71.5 ± 24.9 ml. AO_forward_ measured by phase-contrast cine CMR was available in 24 patients (68.6%) and was highly correlated with RVSV (*R* ^2^ = 0.97, *p* < 0.001). Agreement between the two methods was also excellent (difference: −0.1 ml; 95%-confidence interval −8.7 to 8.5 ml, *p* = 0.92 vs 0). All analyses were performed using RVSV (*n* = 35) as the reference stroke volume. The same analyses were repeated in the subset of patients (*n* = 24) with AO_forward_ as the reference stroke volume, and the corresponding results were indicated for comparison.

### Reproducibility of prolapsed volume measurement

Intra-observer analysis of prolapsed volume measurement (*n* = 35) indicated a good agreement: mean difference: −0.4 ml (95%-confidence interval: −3.6 to 2.8 ml). The inter-observer analysis indicated a mean difference of −2.6 ml (95%-confidence interval: −8.8 to 3.6 ml). In the subgroup of patients assessed with a 1.5 T scanner (*n* = 26), the mean differences were 0.1 ml (95%-confidence interval: −2.3 to 2.5 ml) and −2.3 ml (−8.7 to 4.1 ml) for intra- and inter-observer analysis, respectively. In the subgroup of patients assessed with a 3.0 T scanner (*n* = 9), the mean differences were −1.8 ml (95%-confidence interval: −5.5 to 2.0 ml) and −3.6 ml (−9.2 to 2.0 ml) for intra- and inter-observer analysis, respectively.

### Agreement between reference RVSV and LVSV in patients with no MR

**Fig. 3:**
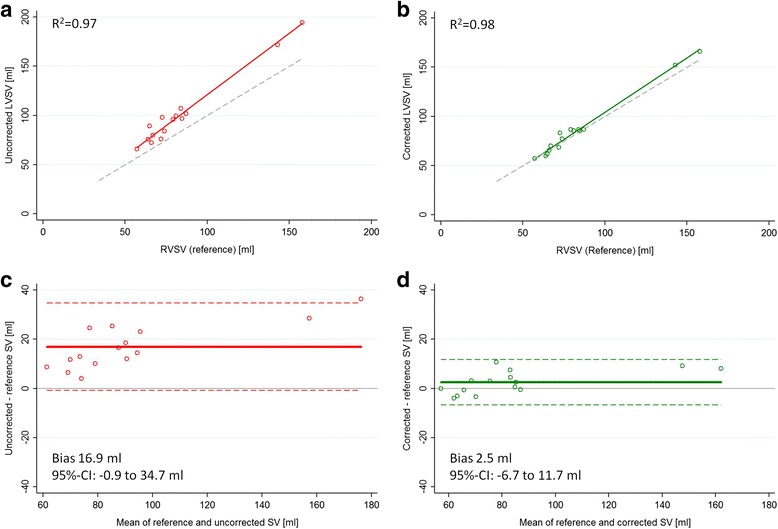
Comparison of LVSV and reference SV in 15 patients with no significant mitral regurgitation. Regression (*top panels*) and Bland-Altman (*bottom panels*) plots comparing LVSV and the reference stroke volume (RVSV) in 15 patients with no significant mitral regurgitation. With the uncorrected method, the LVSV was higher than the reference stroke volume (*left hand panels*), while with the corrected method, no significant difference was measured (*right hand panels*). **a** Correlation between uncorrected and reference SV in patients with no mitral regurgitation **b** Correlation between corrected and reference SV in patients with no mitral regurgitation (MR) **c** Agreement between uncorrected and reference SV in patients with no MR **d** Agreement between corrected and reference SV in patients with no MR

Fifteen patients (42.9%) had no or only trace MR and their reference RVSV was 83.6 ± 28.6 ml. The mean prolapsed height was 7.6 ± 2.1 mm and the prolapsed volume was 14.4 ± 7.0 ml. The LVSV_corr_ was significantly lower than the LVSV_uncorr_ (86.1 ± 31.6 ml vs 100.6 ± 35.8 ml, *p* < 0.0001). In the patients with no MR, the LVSV_uncorr_ exceeded the true reference RVSV by 16.9 ± 9.1 ml (*p* = 0.0007 vs 0), while a non-significant 2.5 ± 4.7 ml overestimation was observed with the LVSV_corr_ (*p* = 0.11 vs 0; Fig. 3). In 8 of these patients, AO_forward_ was available as reference stroke volume and a similar overestimation of AO_forward_ was found with LVSV_uncorr_ (mean difference 18.3 ± 11.8 ml, *p* = 0.01 vs 0) but not with LVSV_corr_ (mean difference 4.8 ± 6.6 ml, *p* = 0.16 vs 0).

### Impact of prolapse severity on MR severity grading

**Fig. 4:**
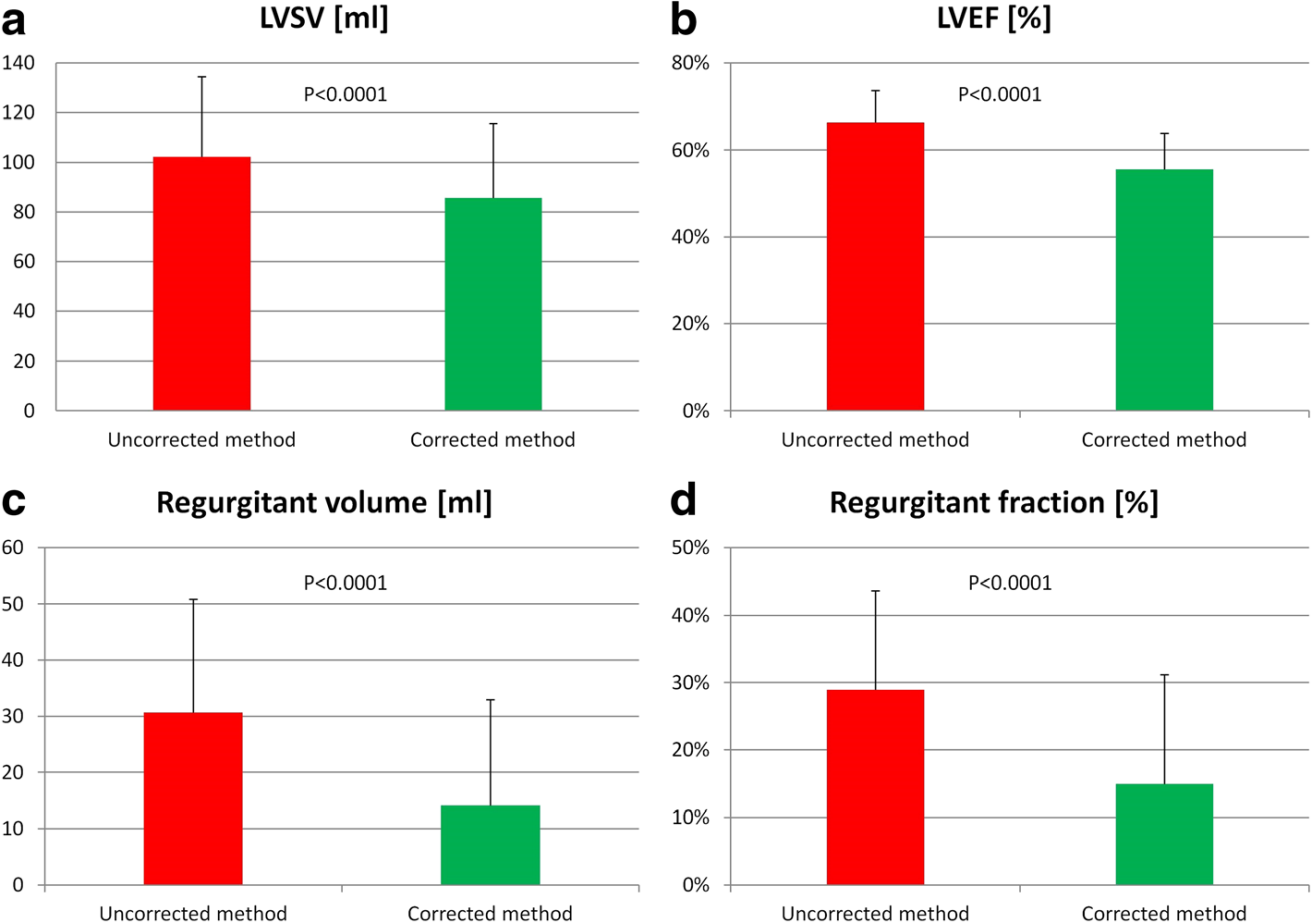
Impact of end-systolic volume correction on the LV function and MR severity assessment. The left ventricular stroke volume (LVSV, panel **a**), LV ejection fraction (LVEF, panel **b**), regurgitant volume across the mitral valve (panel **c**) and mitral regurgitant fraction (panel **d**) all appear significantly higher when measured with the uncorrected method

**Fig. 5:**
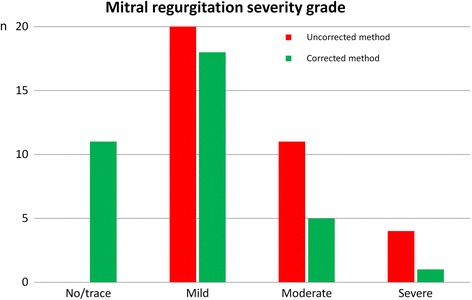
Impact of end-systolic volume correction on MR severity grading. The uncorrected method indicated a 1 grade higher MR severity than the corrected method in 66% of the patients

Over the whole cohort (*n* = 35), the prolapsed height correlated linearly with the difference between uncorrected, and the corrected stroke volume (*r* ^2^ = 0.66, *p* < 0.0001). The uncorrected and corrected method provided significantly different results for LVSV (102.2 ± 32.3 vs 85.7 ± 30.2 ml, respectively, *p* < 0.0001), LVEF (66.4 ± 7.3 vs 55.6 ± 8.3%, respectively, *p* < 0.0001), regurgitant volume (30.7 ± 20.2 vs 14.1 ± 18.8 ml, respectively, *p* < 0.0001) and regurgitant fraction (29.0 ± 14.7 vs 14.9 ± 16.3%, respectively, *p* < 0.0001) (Fig. 4). Accordingly, the distribution of the patients among the four classes of MR severity (none or trace/mild/moderate/severe) was significantly different for the uncorrected and the corrected methods (0/20/11/4 vs 11/18/5/1, respectively, *p* < 0.001). Overall, the grade of MR severity was concordant for the two methods in only 12 patients (34%), as the uncorrected method overestimated MR severity by 1 grade in 23 (66%) patients (Fig. 5). In the subgroup of patients known for having no MR (*n* = 15), CMR suggested the presence of a mild MR in all patients when the uncorrected method was used (with a mean regurgitant volume of 16.9 ± 9.1 ml and a mean regurgitant fraction of 16.3 ± 5.9%). With the corrected method, 11 out of the 15 patients were correctly classified as having no significant MR. In 4 patients, MR grade was still overestimated by one grade despite low regurgitant volumes (mean regurgitant volume of 7.9 ± 2.6 ml, mean regurgitant fraction of 8.2 ± 3.4%).

Among the subset of patients where AO_forward_ was available (*n* = 24), the uncorrected method resulted in a similar overestimation of the LVSV (104.8 ± 33.1 vs 88.0 ± 31.1 ml, *p* < 0.0001), the LVEF (66.4 ± 7.9 vs 55.8 ± 8.7%, *p* < 0.0001), the regurgitant volume (35.1 ± 22.4 vs 18.4 ± 20.9 ml, *p* < 0.0001) and the regurgitant fraction (32.2 ± 16.2 vs 19.3 ± 17.6%, *p* < 0.0001). MR grade severity was concordant between the corrected and the uncorrected methods in only 10 (41.7%) patients, and an overestimation by one grade was observed in 14 (58.3%) patients.

## Discussion

In the MVP population, the present study suggests a correction of the LVSV by the prolapsed volume, i.e. by the volume “contained” within the prolapsing mitral valve leaflets, impacts on the quantification of MR. This correction reduced the MR severity by one grade in 66% of patients. In the case of a prominent MVP, the mitral annulus is not an adequate landmark to separate the LV from the LA volume in systole, as the systolic billowing of the mitral valve into the LA creates a space in the LA that is occupied by the LV blood pool. This part of the LV blood pool (i.e. the prolapsed volume) is not crossing through the incompetent mitral valve in systole. i.e. this volume does not contribute to the regurgitant volume of the prolapsing mitral valve, and should therefore be corrected for. Accordingly, in the situation of MVP, the proximal border of the LV blood pool is the plane of the mitral valve leaflets, not the mitral annulus. As the mitral valve is a thin structure, it is difficult to recognize on short-axis cine slices of the LV and we propose a method using the 3 long-axis orientations to correct the standard LVESV for the prolapsed volume. In the patients known for having no or only trace MR, a better agreement was found between the LVSV_corr_ and the reference RVSV with this correction for the prolapsed volume. Over the whole cohort of patients with MVP, this correction resulted in a difference in MR severity quantification by 1 grade in almost two thirds of the patients. In addition, with this correction, LV ejection fraction was also significantly lower, reflecting the ineffective pump function caused by the systolic displacement of the mitral valve plane into the LA.

With regard to the need to undergo MV surgery, one single recent study proposed a validated cut-off value for Reg_Vol_ and RF with CMR [10], and no general consensus exists regarding the most appropriate CMR quantification technique. Several CMR approaches have been used including the comparison of the LVSV with the RVSV [13], LVSV with the Ao_forward_ by phase-contrast cine imaging [15, 17], trans-mitral forward flow with Ao_forward_ [12, 23], or by direct measurement of the regurgitant orifice area [24]. However, these methods were not specifically validated for MVP patients and they seem not to be interchangeable, as they can differ in quantitative measures of regurgitation as well as with respect to inter-observer reproducibilities [25, 26]. In case of MVP, the present study suggests that MR quantification has to be adapted to the mechanism of MR to minimize the risk of misclassification. Our population essentially consisted of patients with severe MVP and the mean prolapsed volume was as large as 16 ml, indicating, theoretically, that the calculated regurgitant volume was systematically overestimated by 16 ml. This volume is by no means negligible as it represents one fourth of the cut-off value, which defines a severe MR [21]. Not surprisingly, this systematic error resulted in an overestimation of MR severity by one grade in almost two thirds of the MVP patients.

From a clinical perspective, mitral valve surgery is indicated in patients with severe MR, who present with symptoms or evidence of LV dilatation or dysfunction [27], but there is evidence suggesting that early surgery of severe MR before the onset of symptoms or LV dysfunction might be beneficial in terms of long term prognosis if the morphology of the valve allows a successful durable repair [28]. Mitral valve repair is more challenging in the presence of severe bileaflet MVP due to the diffuse nature of the morphologic changes and valve replacement may be the only surgical option for some patients [6, 29]. Accordingly, a correct quantification of MR is crucial to avoid unnecessary operations. The consequences of early surgery in patients with non-severe MR could include a higher risk of re-do surgery later in life and it would increase morbidity in the patients for whom mitral valve repair was not possible resulting in mitral valve replacement. The appropriate timing of the first surgical repair is of particular importance for patients with MVP as they typically represent a relatively young population, where the perspective of re-do surgery exists despite excellent long-term results of surgical mitral valve repair [30]. Finally, a systematic overestimation of MR severity may generate additional unnecessary follow-up visits and create anxiety to the patient. As an illustration, by using the cut-off values prospectively validated by Myerson et al. (RegVol >55 ml or a RF >40%) [10], and applying the standard uncorrected method, 9 (26%) patients would be considered as potential candidates for surgery versus only 4 (11%, *p* = 0.002) when using the proposed correction method, which considers the prolapsed volume, i.e. the severity of the valve prolapse.

The limitations of this study include the relatively small number of participating patients and the specific mechanism of valve disease investigated, which is not representative for other types of MR. The retrospective design of this study may have selected patients with a more severe degree of MVP (mean prolapsed height was 8.1 mm) and the risk of MR misclassification may be less important for patients with milder degrees of MVP. We are therefore not able to recommend a cut-off value of prolapsed height above which a LVSV correction might be warranted. In this study, the prolapsed volume has been estimated and not directly measured. The geometric assumptions associated with our correction strategy might have induced some variability in the measurements. However, the proposed correction method can be easily applied to conventional CMR protocols which typically include a LV short-axis stack and 3 long-axis cine acquisitions. In this regard, the development of a time-efficient truly three-dimensional technique to accurately measure the prolapsed volume might be of value. Finally no direct comparison with echocardiographic MR quantification was performed in this cohort, as the aim of this study was not to compare the performance of two different imaging methods, but to highlight a potential methodological bias induced by MVP when the quantification of MR severity is performed with a generally accepted standard CMR method.

## Conclusion

When applying the standard method of discs to measure LV volumes in patients with MVP, the blood volume contained within the valve prolapse is not considered to be part of the LV volume in systole. Consequently, in a severe form of bileaflet prolapse, this may lead to a significant underestimation of the true end-systolic volume and thus, overestimation of MR severity. For these patients, we suggest to correct the LVSV for the prolapsed volume, as it allowed a reclassification of MR severity to a milder grade in two thirds of the MVP patients.

## Abbreviations

AO_forward_: Aortic forward flow
CMR: Cardiac magnetic resonance
FOV: Field of view
LA: Left atrium
LV: Left ventricle
LVEDV: Left ventricular end-diastolic volume
LVESV: Left ventricular end-systolic volume
LVESV_corr_: Left ventricular end-systolic volume (corrected for prolapsed volume)
LVESV_uncorr_: Left ventricular end-systolic volume (Simpson’s method)
LVSV: Left ventricular stroke volume
MR: Mitral regurgitation
MVP: Mitral valve prolapse
PISA: Proximal isovelocity surface area
Regvol: Regurgitant volume
RF: Regurgitant fraction
RVSV: Right ventricular stroke volume
SV: Stroke volume
TE: Echo time
TR: Repetition time
VENC: Velocity encoding
